# Influencing factors of stroke in patients with type 2 diabetes: A systematic review and meta-analysis

**DOI:** 10.1371/journal.pone.0305954

**Published:** 2024-06-24

**Authors:** Mengjiao Zhao, Yongze Dong, Luchen Chen, Huajuan Shen

**Affiliations:** 1 School of Nursing, Zhejiang Chinese Medical University, Hangzhou, 310053, China; 2 Department of Nursing, Zhejiang Provincial People’s Hospital (Affiliated People’s Hospital), Hangzhou Medical College, Hangzhou, 310014, China; Universita Politecnica delle Marche, ITALY

## Abstract

**Background:**

Stroke stands as a significant macrovascular complication among individuals with Type 2 diabetes mellitus (T2DM), often resulting in the primary cause of mortality and disability within this patient demographic. Presently, numerous studies have been conducted to investigate the underlying causes of stroke in individuals with T2DM, yet the findings exhibit inconsistencies.

**Objective:**

This paper aims to consolidate and summarize the available evidence concerning the influential factors contributing to stroke among patients diagnosed with T2DM.

**Methods:**

We conducted a comprehensive search across multiple databases, including Cochrane Library, PubMed, Web Of Science, Embase, China Biology Medicine (CBM), China National Knowledge Infrastructure (CNKI), Wanfang and Weipu up to August 2023. Google Scholar was also searched to retrieve gray literature. We calculated odds ratios (OR) and 95% confidence intervals (CI) using Stata software.

**Results:**

Our analysis encompassed 43 observational studies, exploring factors across sociodemographic, biochemical, complications, and hypoglycemic agent categories. The findings identified several risk factors for stroke in patients with T2DM: age, gender, T2DM duration, hypertension, body-mass index (BMI), smoking, Glycated hemoglobin (HbA1c), estimated Glomerular Filtration Rate (eGFR), albuminuria, Triglycerides (TG), Low density lipoprotein cholesterol (LDL-C), Coronary heart disease (CHD), Atrial fibrillation (AF), diabetic retinopathy (DR), Peripheral vascular disease (PVD), and carotid plaque. Conversely, exercise, High density lipoprotein cholesterol (HDL-C), metformin (MET), pioglitazone, and metformin combination therapy emerged as protective factors.

**Conclusion:**

This study underscores the multitude of influencing factors contributing to stroke in people with T2DM patients, among which the microvascular complications of T2DM play an most important role. Therefore, we emphasize the importance of screening for microvascular complications in patients with T2DM. However, due to limitations arising from the number of articles reviewed, there remain areas where clarity is lacking. Further research efforts are warranted to expand upon and reinforce our current findings.

## 1. Introduction

T2DM currently affects an estimated 10.5% (536.6 million) of the global population, a figure expected to escalate to 12.2% (783.2 million) by 2045, as projected by the International Diabetes Federation [1]. This chronic condition predisposes individuals to various macrovascular and microvascular complications, significantly contributing to mortality rates worldwide [2–3]. Stroke, among the prevalent macrovascular complications associated with T2DM, accounted for 6.55 million fatalities in 2019, securing its place as the second leading cause of death globally [4]. Notably, individuals with diabetes face a two to four fold increased risk of stroke compared to their nondiabetic counterparts. Moreover, diabetic patients tend to experience exacerbated post-stroke outcomes and possess a heightened susceptibility to stroke recurrence [5–6].

The occurrence of stroke in T2DM patients results from a convergence of factors including age, gender, hypertension, smoking, dyslipidemia, and more [7–9]. Recent studies indicate a predictive relationship between T2DM microvascular complications such as diabetic nephropathy (DN), DR, diabetic neuropathy, and the likelihood of stroke [10]. Furthermore, emerging evidence recognizes the cardiovascular protective properties of novel hypoglycemic drugs like sodium-glucose cotransporter-2 inhibitors (SGLT-2is) and glucagon-like peptide-1 receptor agonists (GLP-1 RAs) [11]. However, the existing body of research on stroke causation in T2DM patients exhibits variations in focus, population demographics, sample sizes, and consequent disparate findings.

Therefore, we conducted the current systematic review and meta-analysis to review the influencing factors of stroke in patients with T2DM worldwide and explore the strengths of such associations for early identification and prevention of stroke.

## 2. Methods

This review was conducted in accordance with the Preferred Reporting Items for Systematic Reviews and Meta-Analyses (PRISMA) [12]. The study was registered in the "International Prospective Register of Systematic Reviews" (PROSPERO) on November 20, 2023 (CRD42023480426).

### 2.1 Search strategy

Articles were searched on eight electronic databases, including PubMed, Web Of Science, Embasse, Cochrane Library, CBM, CNKI, Weipu and Wanfang database. Gray literature was researched in Google Scholar. We performed the search strategy until August 2023. A combination of MeSH terms and free terms related to “Diabetes Mellitus, Type 2 OR Diabetes Mellitus, Type II OR Type 2 Diabetes Mellitus OR Type 2 Diabetes OR Diabetes, Type 2 OR Diabetes Mellitus, Noninsulin-Dependent OR Diabetes Mellitus, Non Insulin Dependent OR Diabetes Mellitus, Non-Insulin-Dependent OR Non-Insulin-Dependent Diabetes Mellitus”, “Strokes OR Stroke OR Cerebrovascular Accident* OR Cerebrovascular Apoplexy OR Apoplexy, Cerebrovascular OR Vascular Accident, Brain OR Brain Vascular Accident* OR Cerebral Stroke* OR Apoplexy OR Stroke, Acute OR Acute Cerebrovascular Accident OR Hemorrhagic Stroke* OR Ischemic Stroke* OR Acute Ischemic Stroke* OR Thrombotic Stroke* OR Embolic Stroke* OR Cerebral Infarction*”, “risk factors OR risk factor OR influence factor* OR relevant factor*” were used to search. (S1 Table in S1 Appendix)

### 2.2 Inclusion and exclusion criteria

Studies that reported possible influencing factors of stroke in patients with T2DM were selected based on these inclusion criteria: (1) Patient age ≥ 18 years; (2) observational study (case control, cohort and cross-sectional study); (3) studies that provide the OR with 95% CI, or can be calculated with sufficient information. (4) English or Chinese article. Studies were excluded if they were: (1) duplicate literature; (2) case reports, reviews, conference abstracts, systematic reviews; (3) incomplete or unavailable literature.

### 2.3 Quality assessment

Two researchers, Mengjiao Zhao and Luchen Chen, independently evaluated article quality using the Newcastle-Ottawa Scale (NOS) for cohort and case-control studies (score ≥ 7 considered high quality) [13], and the criteria of America Agency for Healthcare Research and Quality (AHRQ) for cross-sectional studies (score ≥ 8 considered high quality) [14]. In the case of uncertainty or disagreement about quality, the article was reviewed by a third researcher, Yongze Dong.

### 2.4 Data extraction

Data extraction was performed by Mengjiao Zhao and Luchen Chen using a standardized form, encompassing details like author, publication year, country, study type, sample size, influencing factors, and adjusted OR with 95% CI for potential confounding variables. Consensus was reached in cases of disagreement through group discussion.

### 2.5 Data synthesis and statistical analysis

Stata 15.1 software facilitated data analysis, computing pooled OR with 95% CI. A significance level of P < 0.05 was applied. Heterogeneity was assessed using Cochran Q and I^2^ statistics, adopting a fixed-effects model in the absence of significant heterogeneity (P > 0.10 and I^2^ ≤ 50%). Otherwise, a random-effects model was employed. Subgroup and sensitivity analyses were performed to explore heterogeneity causes. We analysed subgroups by study area, sample size, type of study design and different classes of influencing factors. Additionally, sensitivity analyses by iteratively removing one study at a time. Egger’s linear regression test gauged publication bias (P > 0.05 indicates no significant publication bias; P < 0.05 suggests publication bias).

## 3. Results

### 3.1 Search results

**Fig 1:**
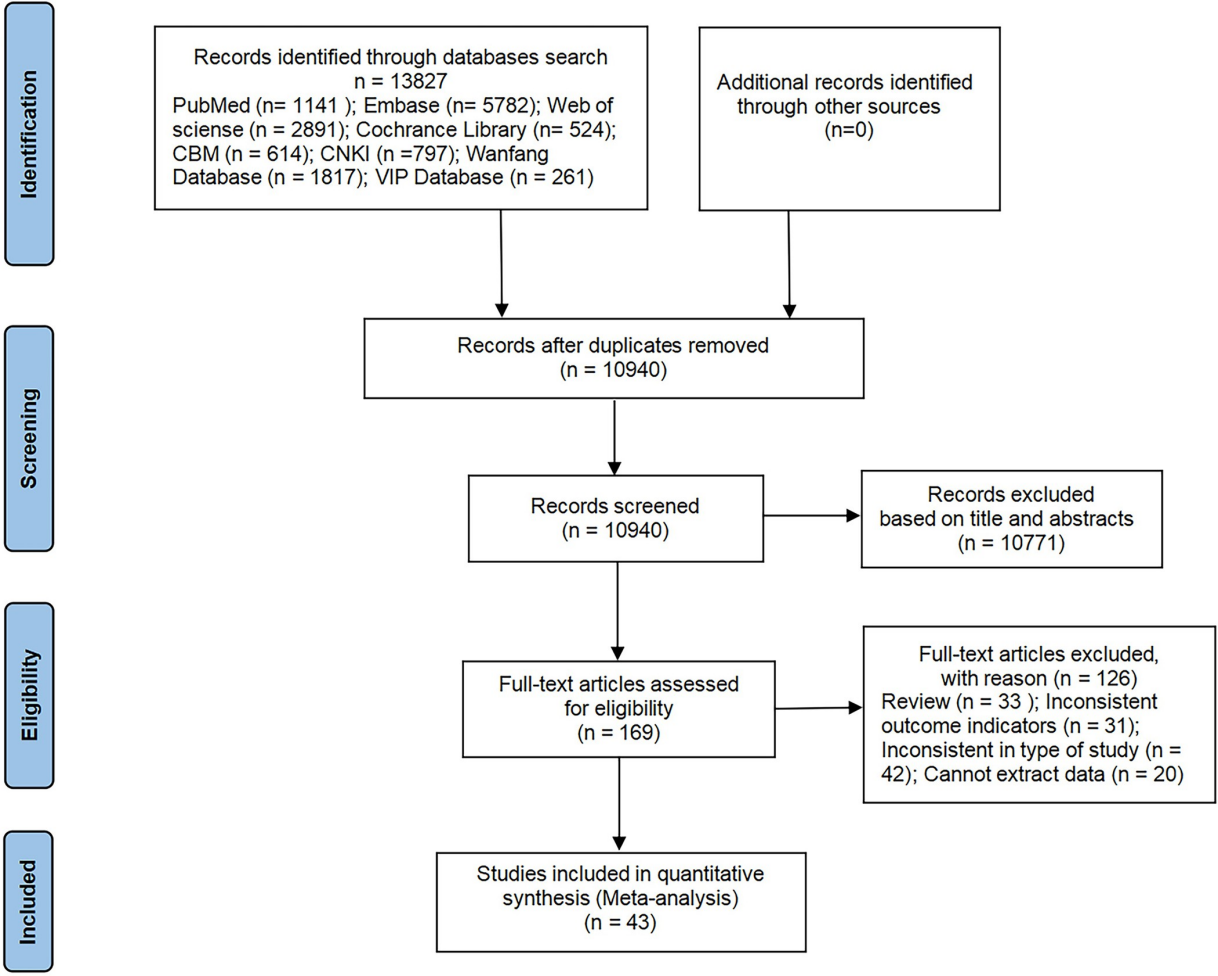
PRISMA flow diagram of study selection and inclusion process.

As shown in Fig 1. In total, 13,827 articles were initially identified, with 2,887 duplicates removed. After screening titles and abstracts, 10,771 papers were excluded. A full-text assessment of 169 studies followed, resulting in the exclusion of 126 ineligible studies. Ultimately, 43 studies met the eligibility criteria for inclusion [15–57].

### 3.2. Characteristics of the included studies

Table 1 showcases the essential features of the 43 incorporated articles, spanning publication years from 2001 to 2023. The studies comprised 28 cohort studies, 8 case-control studies, and 7 cross-sectional studies. Among these, 22 were conducted in developed nations (USA, UK, Australia, Denmark, New Zealand, Spain, Korea, Japan) and 21 in developing countries (China, Saudi Arabia). The sample sizes varied from 96 [30] to 1,297,131 [29], totaling 2,730,010 participants. The quality assessment showed a literature quality assessment score of 7–9 for cohort and case-control studies and 8–10 for cross-sectional studies. (S2-S4 Table in S1 Appendix)

**Table 1:** Characteristics of the included studies.

First Author	Year of study	Country	Study Type	Sample Size	Influencing factors	Quality Score
**Yu**	**2023**	**China**	**cohort**	**2625**	**29**	**8**
**Kim**	**2022**	**Korea**	**cohort**	**181591**	**8,13**	**9**
**Xu**	**2022**	**China**	**cross-sectional**	**1119**	**1,2,3,4,5,6,8**	**8**
**Wu**	**2022**	**China**	**cohort**	**185813**	**1,2,11,12**	**9**
**Zhou**	**2022**	**China**	**cohort**	**174935**	**31**	**9**
**Wu2**	**2022**	**China**	**case-control**	**445**	**5,19,20,25**	**9**
**Lin**	**2022**	**China**	**cohort**	**232101**	**32**	**8**
**Iwase**	**2021**	**Japan**	**cohort**	**4875**	**1,2,4,7,8**	**8**
**Isaman**	**2021**	**USA**	**cohort**	**3575**	**1,12**	**8**
**He**	**2021**	**China**	**cross-sectional**	**18013**	**5,8,13,14**	**9**
**Salinero**	**2021**	**Spain**	**cohort**	**2980**	**1,7,9,10,21,22,23,26**	**9**
**Modjtahedi**	**2021**	**Spain**	**cohort**	**77376**	**21**	**9**
**Ha**	**2021**	**Korea**	**case-control**	**128171**	**30**	**9**
**Su**	**2020**	**China**	**case-control**	**2169**	**1,2,3,5,6**	**8**
**Shi**	**2020**	**China**	**cross-sectional**	**4335**	**1,6,7,10,27,28**	**10**
**Drinkwater**	**2020**	**Australia**	**cohort**	**1473**	**21,22,23**	**9**
**Kim**	**2020**	**Korea**	**cohort**	**1297131**	**3,8,11,21**	**9**
**Adderley**	**2020**	**UK**	**cohort**	**14117**	**16**	**9**
**Fangel**	**2020**	**Denmark**	**cohort**	**69532**	**22**	**9**
**Hung**	**2020**	**China**	**cohort**	**13078**	**31**	**9**
**Geng**	**2019**	**China**	**case-control**	**321**	**27,28**	**9**
**Alramadan**	**2019**	**Saudi Arabia**	**cross-sectional**	**1111**	**2,5,17.**	**8**
**Niwa**	**2019**	**Japan**	**case-control**	**816**	**1,5,24**	**8**
**Komi**	**2018**	**Japan**	**cohort**	**1606**	**5**	**9**
**Chan**	**2018**	**China**	**cohort**	**26742**	**34**	**9**
**Hsu**	**2018**	**China**	**cohort**	**14306**	**34**	**7**
**Noh**	**2017**	**Korea**	**cohort**	**2006**	**1,4,5,7**	**8**
**0U**	**2017**	**China**	**cohort**	**113051**	**34**	**8**
**Zimmerman**	**2017**	**USA**	**cohort**	**14117**	**33**	**9**
**Sun**	**2017**	**China**	**cross-sectional**	**1401**	**7**	**8**
**Ye**	**2016**	**China**	**case-control**	**544**	**1,3,25**	**9**
**Zghebi**	**2016**	**UK**	**cohort**	**13576**	**34**	**8**
**Liu**	**2014**	**China**	**cohort**	**8306**	**1,9,18**	**8**
**Wang**	**2014**	**USA**	**cohort**	**28391**	**7**	**8**
**Li**	**2014**	**UK**	**cohort**	**21998**	**1,8,10,24,15**	**9**
**Cheng**	**2014**	**China**	**cohort**	**14856**	**29**	**8**
**Marfella**	**2013**	**Australia**	**cohort**	**464**	**23**	**8**
**Bouchi**	**2012**	**Japan**	**cross-sectional**	**786**	**24**	**10**
**Nomura**	**2010**	**Japan**	**cross-sectional**	**217**	**1**	**8**
**Elley**	**2008**	**New Zealand**	**cohort**	**48444**	**4**	**9**
**Xu**	**2004**	**China**	**case-control**	**96**	**3,4,5,9**	**7**
**Gillett**	**2003**	**Australia**	**cohort**	**1181**	**3,9.21.22.25**	**9**
**Meng**	**2001**	**China**	**case-control**	**220**	**5,11,21**	**7**

Note: Influencing factors:1 = Age; 2 = Gender; 3 = Course of T2DM; 4 = HbA1c; 5 = Hypertension; 6 = BMI; 7 = eGFR; 8 = smoking; 9 = Total cholesterol (TC); 10 = TG; 11 = CHD; 12 = Congestive heart failure; 13 = Exercise; 14 = Sleep duration; 15 = Alcohol abuse; 16 = Obstructive sleep apnea; 17 = Lower level of education; 18 = Central obesity; 19 = Free triiodothyronine; 20 = Cerebral artery stenosis degree; 21 = AF; 22 = Albuminuria; 23 = DR; 24 = PVD; 25 = Carotid Plaque; 26 = Diabetic neuropathy; 27 = HDL-C; 28 = LDL-C; 29 = MET; 30 = Pioglitazone; 31 = Sulphonylureas (SU); 32 = SGLT2-i; 33 = GLP-1RA; 34 = MET combination therapy (MET + thiazolidinediones, MET + alpha-glucosidase inhibitors, MET + dipeptidyl peptidase-4 inhibitors).

### 3.3. Meta-analysis for influencing factors

**Fig 2:**
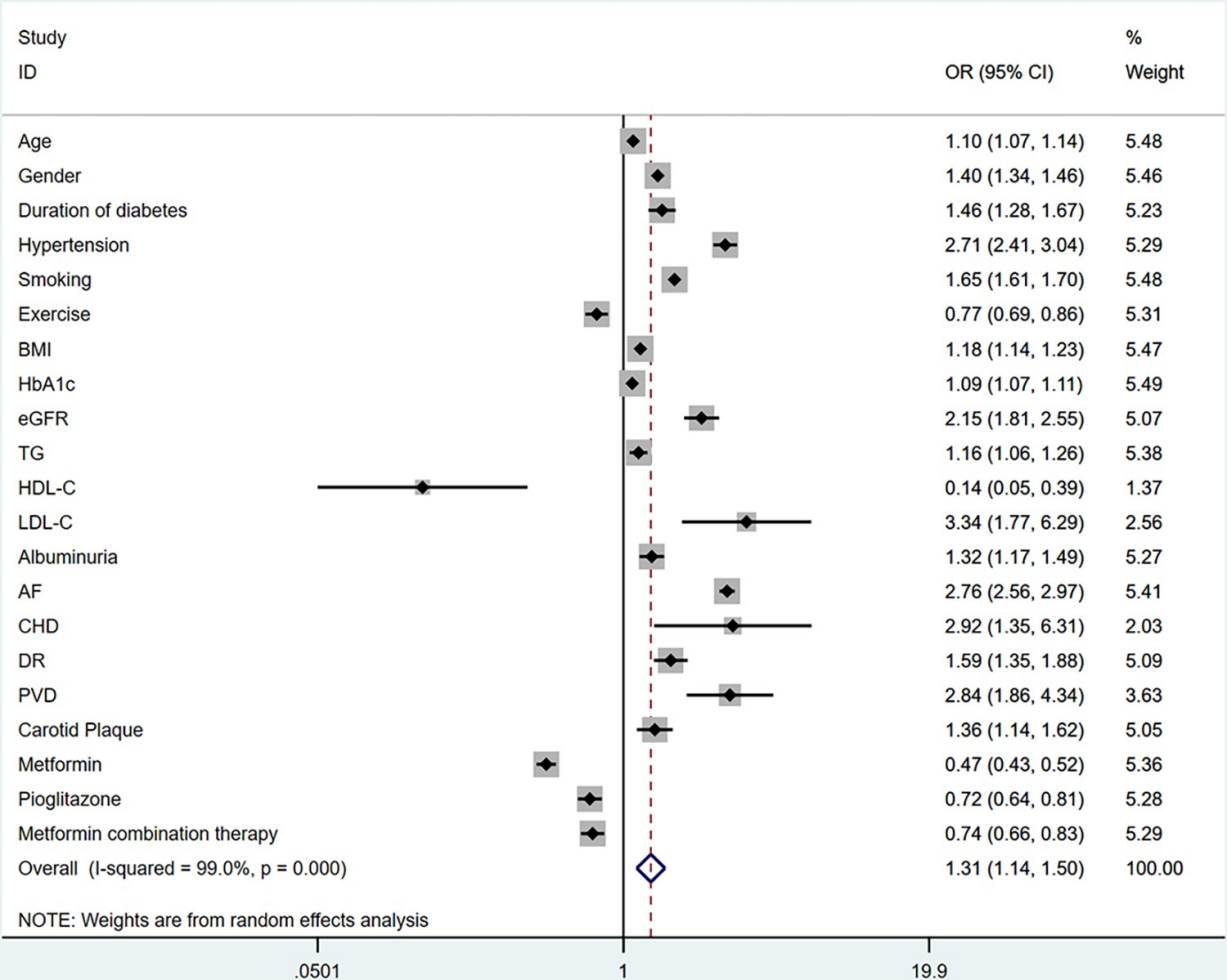
The forest plot of all influencing factors associated with stroke in patients with T2DM.

The meta-analysis encompassed 22 influencing factors categorized into sociodemographic factors, biochemical factors, complications, and hypoglycemic agents. Among the sociodemographic factors are age [15–26], gender [15,17,20,27–28], course of T2DM [15,20,23,29,31], hypertension [15,20–22,28,30,32–35], BMI [15,19–20,33], smoking [15,17,24,29,33,36] and exercise [29,33,36]; Biochemical indexes are HbA1c [15,17,22,30,37], eGFR [16,19,22,38–39], TC [16,25,30–31], TG [16,19,24], Albuminuria [16,31,40–41], HDL-C [19,42] and LDL-C [19,42]; Complications are CHD [27,29,35], AF [16,29,31,35,41,43], DR [16,41,44], PVD [21,24,45] and carotid plaque [23,32], as well as hypoglycemic agents are MET [46–47], pioglitazone [48–49] and MET combination therapy [50–53]. Among the four categories, the pooled OR of complications was the highest (2.09, 95% CI: 1.44–3.05), followed by sociodemographic factors (1.37, 95% CI: 1.14–1.65) and biochemical factors (1.28, 95% CI: 1.05–1.57), while the hypoglycemic agents was the lowest (0.63, 95% CI: 0.46–0.85), which was a protective factor. The details are shown in Table 2. In addition, the forest plot containing all the influencing factors is shown in Fig 2.

**Table 2:** The results of factors associated with stroke in the patients with T2DM.

Factor	Reference (n)	I^2^ (%)	P(Q)	Model	OR	95%CI	P (Value)	Egger’s Test
**Sociodemographic factors**	**22**				**1.37**	**1.14–1.65**		
**Age**	**12**	**86**	**<0.001**	**Random**	**1.10**	**1.06–1.13**	**<0.001**	**0.000**
**Gender**	**5**	**17**	**0.310**	**Fixed**	**1.40**	**1.34–1.46**	**<0.001**	**0.095**
**Duration of diabetes**	**6**	**73**	**0.002**	**Random**	**1.46**	**1.28–1.67**	**<0.001**	**0.131**
Hypertension*	**9**	**0**	**0.450**	**Fixed**	**2.71**	**2.41–3.04**	**<0.001**	**0.361**
Smoking*	**5**	**0**	**0.820**	**Fixed**	**1.65**	**1.60–1.69**	**<0.001**	**0.885**
**Exercise**	**3**	**84**	**0.002**	**Random**	**0.77**	**0.69–0.86**	**<0.001**	**0.803**
**BMI**	**4**	**12**	**0.330**	**Fixed**	**1.18**	**1.14–1.23**	**<0.001**	**0.261**
**Biochemical factors**	**17**				**1.28**	**1.05**–1.**57**		
**HbA1c**	**5**	**97**	**<0.001**	**Random**	**1.09**	**1.07–1.11**	**<0.001**	**0.157**
eGFR*	**4**	**0**	**0.550**	**Fixed**	**2.15**	**1.81–2.55**	**<0.001**	**0.241**
**TC**	**4**	**79**	**0.002**	**Random**	**0.90**	**0.59–1.37**	**0.616**	**0.392**
**TG**	**3**	**0**	**0.430**	**Fixed**	**1.16**	**1.06–1.26**	**<0.001**	**0.059**
**HDL-C**	**2**	**83**	**0.010**	**Random**	**0.14**	**0.05–0.39**	**<0.001**	**NA**
**LDL-C**	**2**	**73**	**0.060**	**Random**	**3.41**	**1.81**–**6.41**	**<0.001**	**NA**
**Albuminuria**	**3**	**7**	**0.343**	**Fixed**	**1.32**	**1.17–1.49**	**<0.001**	**0.016**
**Complication**	**13**				**2.09**	**1.44–3.05**		
**AF**	**6**	**19**	**0.290**	**Fixed**	**2.76**	**2.56–2.97**	**<0.001**	**0.190**
**CHD**	**3**	**98**	**<0.001**	**Random**	**2.92**	**1.35–6.3**	**0.006**	**0.420**
**DR**	**3**	**0**	**0.470**	**Fixed**	**1.59**	**1.35–1.88**	**<0.001**	**0.381**
**PVD**	**2**	**0**	**0.520**	**Fixed**	**2.84**	**1.86–4.34**	**<0.001**	**0.620**
**Carotid Plaque**	**2**	**27**	**0.240**	**Fixed**	**1.36**	**1.14–1.62**	**<0.001**	**NA**
**Hypoglycemic Agents**	**8**				**0.63**	**0.46–0.85**		
**Metformin**	**2**	**0**	**0.390**	**Fixed**	**0.47**	**0.43–0.52**	**<0.001**	**NA**
**Pioglitazone**	**2**	**0**	**0.350**	**Fixed**	**0.72**	**0.64–0.81**	**<0.001**	**NA**
Metformin combination therapy*	**4**	**0**	**0.480**	**Fixed**	**0.74**	**0.66–0.83**	**<0.001**	**0.178**

* After sensitivity analysis, the studies with great influence on the results were excluded.

NA: not applicable.

#### 3.3.1 Sociodemographic factors

A study of 22 articles exploring the correlation between sociodemographic factors and stroke in T2DM patients highlighted age, hypertension, and duration of T2DM as frequently studied factors. Notably, hypertension demonstrated the highest pooled OR (2.71, 95% CI: 2.41–3.04), followed by smoking (1.65, 95% CI: 1.60–2.97). Additionally, factors such as longer T2DM duration (1.46, 95% CI: 1.28–1.67), male gender (1.40, 95% CI: 1.34–1.46), BMI (1.18, 95% CI: 1.14–1.23), and age (1.10, 95% CI: 1.06–1.13) were associated with higher stroke risk. Conversely, exercise exhibited a protective effect (0.77, 95% CI: 0.69–0.86).

#### 3.3.2 Biochemical factors

Among 17 articles investigating biochemical factors, LDL-C exhibited the highest pooled OR (3.41, 95% CI: 1.81–6.41), followed by eGFR (2.15, 95% CI: 1.81–2.55) and albuminuria (1.32, 95% CI: 1.17–1.49). Other factors, including TG (1.16, 95% CI: 1.06–1.26) and HbA1c (1.09, 95% CI: 1.07–1.11), were identified as risk factors for stroke, whereas HDL-C (0.14, 95% CI: 0.05–0.39) appeared protective. However, TC (0.90, 95% CI: 0.59–1.37) was not significantly associated with stroke in T2DM patients.

#### 3.3.3 Complications

The analysis of 13 studies exploring complications and stroke revealed CHD (2.92, 95% CI: 1.35–6.30), PVD (2.84, 95% CI: 1.86–4.34), and AF (2.76, 95% CI: 2.56–2.97) as factors with the highest pooled OR. DR(1.59, 95% CI: 1.35–1.88) and carotid plaque (1.36, 95% CI: 1.14–1.62) were also linked to increased stroke risk.

#### 3.3.4 Hypoglycemic agents

Exploring hypoglycemic agents across 8 articles, MET combination therapy (0.74, 95% CI: 0.66–0.83) and pioglitazone (0.72, 95% CI: 0.64–0.81) showed similar pooled OR, indicating a protective effect. In contrast, MET alone exhibited the lowest pooled OR (0.47, 95% CI: 0.43–0.52).

#### 3.3.5 Other factors

In addition to the factors noted above, several variables have shown significant associations with stroke occurrences in patients with T2DM. These factors include central obesity [25] (2.07, 95% CI: 1.39–3.09), inadequate sleep duration [33] (< 6h/ day: 1.44, 95% CI: 1.20–1.73; > 8h/ day: 1.22, 95% CI: 1.05–1.42), obstructive sleep apnea [54] (1.57, 95% CI: 1.27–1.94), lower educational attainment [28] (2.60, 95% CI: 1.20–5.80), alcohol misuse [24] (2.60, 95% CI: 1.20–5.80), degree of cerebral artery stenosis [32] (4.77, 95% CI: 2.60–9.81), diabetic neuropathy [16] (1.73, 95% CI: 1.14–2.64), congestive heart failure [18] (2.08, 95% CI: 1.26–3.42), and SU [55] (as compared to MET: 3.23, 95% CI: 3.01–3.45). Furthermore, Free triiodothyronine [32] (0.36, 95% CI: 0.20–0.64), SGLT2-i [56] (0.85, 95% CI: 0.82–0.88), and GLP-1RA [57] (0.82, 95% CI: 0.74–0.91) have demonstrated negative correlations.

#### 3.3.6 Subgroup analysis

**Fig 3:**
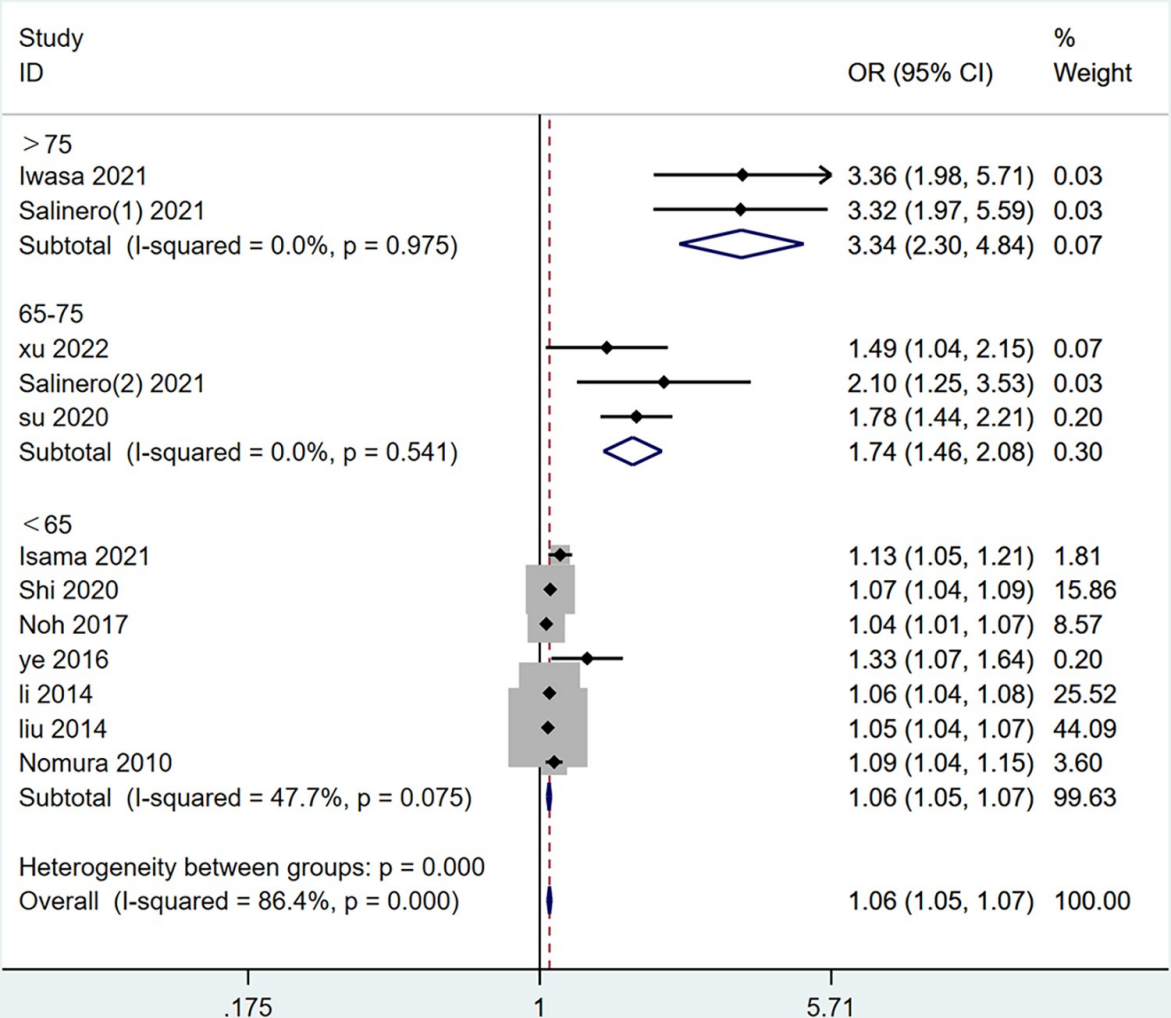
The forest plot of subgroup analysis for age.

**Fig 4:**
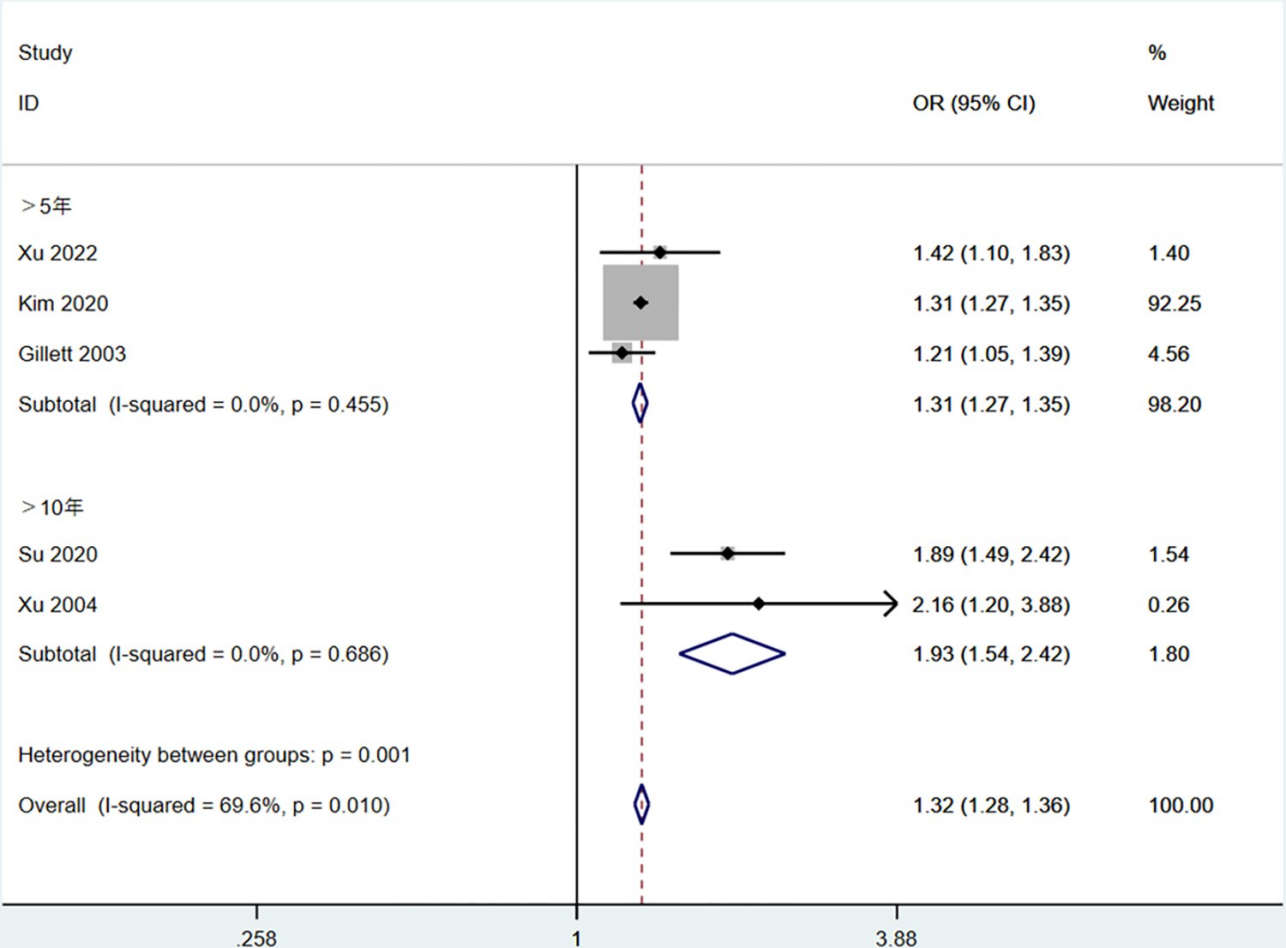
The forest plot of subgroup analysis for the duration of T2DM.

**Fig 5:**
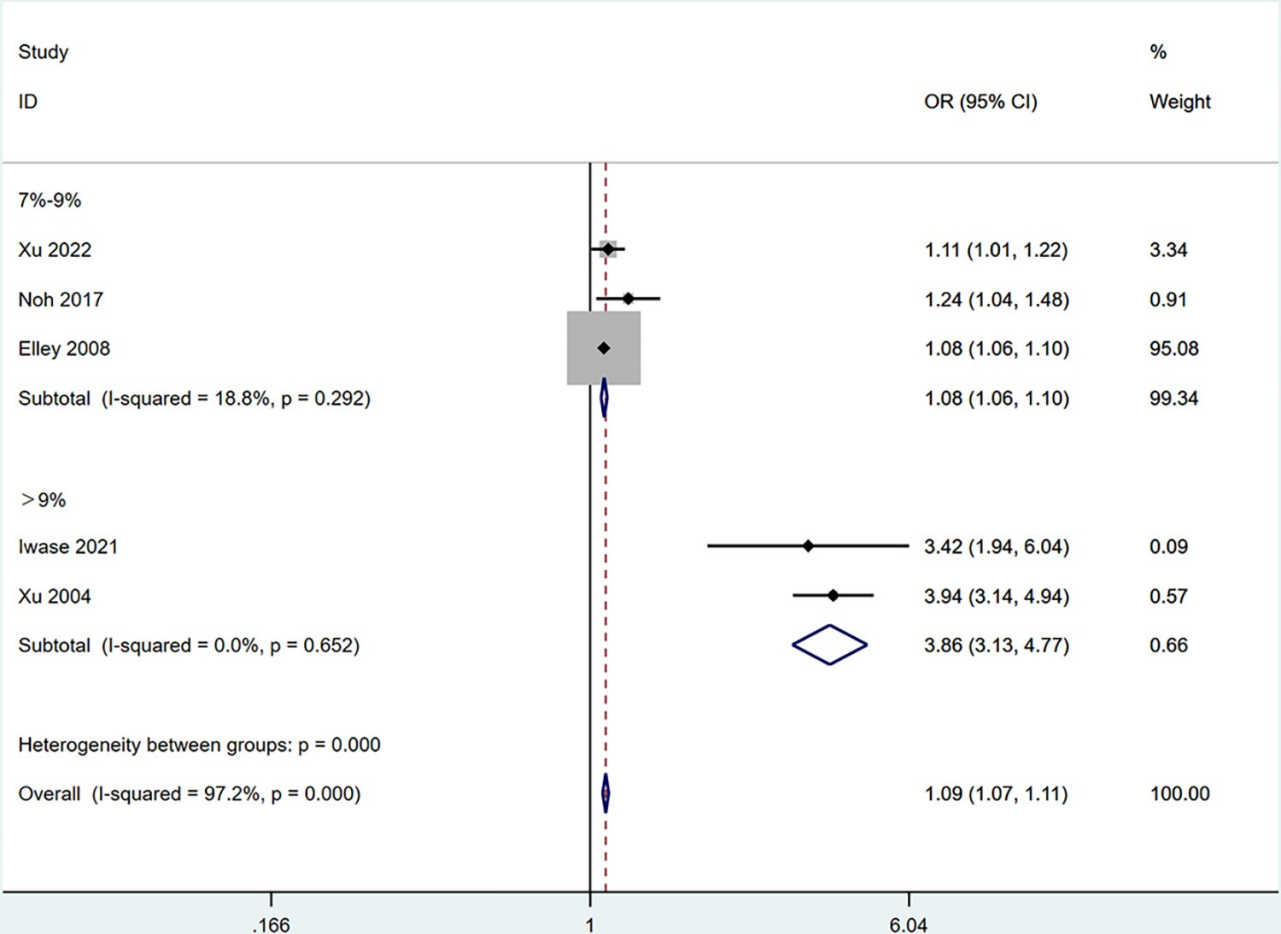
The forest plot of subgroup analysis for HbA1c.

Subgroup analysis was performed on five factors exhibiting high heterogeneity, excluding HDL-C and LDL-C due to the limited number of included articles. These factors comprised age, HbA1c, duration of T2DM, exercise, TC, and CHD. Age was categorized into three subgroups: >75 years old, 65–75 years old, and <65 years old (Fig 3). The duration of T2DM was divided into two subgroups, with exclusion of “Ye 2016” [23] due to unclear reporting: >5 years and >10 years (Fig 4). HbA1c was stratified into two subgroups: 7%-9% and >9% (Fig 5). Sensitivity analyses were employed for subgroups exhibiting persistent high heterogeneity, and specifics are outlined in Table 3.

**Table 3:** The results of subgroup analysis.

Factor	Reference (n)	I^2^ (%)	P (Q)	Model	OR	95%CI	P (Value)	Egger’s Test
**Age**								
**>75**	**2**	**0**	**0.980**	**Fixed**	**3.34**	**2.30–4.84**	**<0.001**	**NA**
65–75*	**3**	**0**	**0.540**	**Fixed**	**1.74**	**1.46–2.08**	**<0.001**	**0.933**
**<65**	**7**	**48**	**0.080**	**Random**	**1.06**	**1.05–1.08**	**<0.001**	**0.020**
**Course**								
**>5 years**	**3**	**0**	**0.460**	**Fixed**	**1.31**	**1.27–1.35**	**<0.001**	**0.885**
**>10 years**	**2**	**0**	**0.690**	**Fixed**	**1.93**	**1.54–2.42**	**<0.001**	**NA**
**HbA1c**								
**7%-8.9%**	**3**	**19**	**0.290**	**Fixed**	**1.08**	**1.06–1.10**	**<0.001**	**0.185**
**>9%**	**2**	**0**	**0.652**	**Fixed**	**3.86**	**3.13–4.77**	**<0.001**	**NA**

* After sensitivity analysis, the studies with great influence on the results were excluded.

NA: not applicable.

Furthermore, subgroup analyses for CHD and exercise and TC were conducted based on study type due to data availability. Only two cohort studies were included for exercise and CHD, and three for TC. Despite these subgroup analyses, the pooled OR still demonstrated high heterogeneity (exercise: I^2^ = 66%, p = 0.09; CHD: I^2^ = 99%, p<0.001; TC: I2 = 70%, p = 0.04). Considering the limited number of included articles and substantial heterogeneity, even post the exclusion of one article in the TC analysis, the initial results were adopted.

#### 3.3.7 Sensitivity analysis

To evaluate the robustness of the association results, we performed a sensitivity analysis by iteratively removing one study at a time and recalculating the summary OR.(S1 Fig in S1 Appendix)

Among the influencing factors, the study by “Xu 2022” [15] exhibited notable impacts on hypertension-related heterogeneity. Upon its exclusion, the pooled OR was found to be 2.71 (2.41, 3.04), with a substantial reduction in heterogeneity (I^2^ = 0%, p = 0.45). Similarly, concerning smoking, exclusion of the same study (“Xu 2022” [15]) resulted in a pooled OR of 1.65 (1.60, 1.69), accompanied by a significant decrease in heterogeneity (I^2^ = 0%, p = 0.82). Furthermore, when considering eGFR, exclusion of the study by “Shi 2020” [19] led to a pooled OR of 2.15 (1.81, 2.55), along with a marked reduction in heterogeneity (I^2^ = 0%, p = 0.55). Additionally, the study by “Chan(1) 2018” [51] affected heterogeneity in MET combination therapy; its exclusion notably decreased heterogeneity (I^2^ = 0%, p = 0.48), yielding a pooled OR of 0.74 (0.66, 0.83).

In the sensitivity analysis, the study conducted by “Niwa 2019” [21] significantly impacted the heterogeneity related to Age 65–75 years. Upon its exclusion, the pooled OR was 1.74 (1.46, 2.08), accompanied by a substantial reduction in heterogeneity (I^2^ = 0%, p = 0.54). Apart from these instances, no significant changes in pooled OR were observed for the other influencing factors, indicating the stability and reliability of our results.

#### 3.3.8 Publication bias

Egger’s test was utilized to assess publication bias within the study. Notably, the P-values obtained from Egger’s test were greater than 0.05 for all exposure variables, except for age (P = 0.000), albuminuria (P = 0.016), and age < 65 years (P = 0.020). These outcomes suggest the presence of publication bias specifically in relation to age, albuminuria, and age < 65 years. Further details regarding these findings are available in Table 2 and Table 3.

## 4. Disscussion

### 4.1 Discussion of the main results

This study delved into a comprehensive exploration of stroke risk factors in patients with T2DM across sociodemographic factors, biochemical factors, complications, and hypoglycemic agent categories. Within these categories, 16 (76%) factors were identified as risk indicators, while 5 (24%) were identified as protective factors through meta-analyses.

Our investigation revealed that sociodemographic and biochemical factors have been extensively studied, among these factors, age emerged as a crucial factor influencing stroke risk in T2DM patients. Subgroup analysis revealed an increasing stroke risk with advancing age, consistent with prior research [58] indicating a higher stroke risk among elderly T2DM patients due to declining bodily functions and the prevalence of cardiovascular risk factors like hypertension and microvascular complications. The timing of T2DM diagnosis was inversely linked to cardiovascular risk [59–60], suggesting the need for heightened vigilance among patients diagnosed at younger ages. Gender-based differences in stroke risk presented conflicting findings. While certain studies [61] suggested a higher risk in women, others [62] indicated the opposite. These disparities might relate to cultural and racial variations among study populations. Additionally, hypertension was strongly associated with an increased risk of stroke in T2DM patients, aligning with previous research [60] attributing this to metabolic syndrome, insulin resistance, and related cardiovascular damage [63]. The duration of T2DM proved to be an independent risk factor for stroke [64]. Studies [5] highlighted a consistent increase in stroke risk with prolonged T2DM duration, the risk of stroke increased by 3% per year in patients with T2DM duration ≥10 years, potentially linked to exacerbated atherosclerosis and endothelial dysfunction. Smoking was identified as another contributor to heightened stroke risk in T2DM patients [65], with evidence supporting smoking cessation as a means of reducing this risk, and smoking cessation in patients with T2DM reduces the risk of ischemic stroke by 20% [66]. Moreover, BMI exhibited a linear relationship with cardiovascular disease risk, with every 5-unit BMI increase correlating with a 9% rise in cardiovascular risk [67]. This association was attributed to obesity-related dyslipidemia, promoting insulin resistance and fostering atherosclerosis [68]. However, other studies have pointed out that insulin resistance reduces the incidence and mortality of cardiovascular disease in obese patients. This self-contradictory conclusion suggests that the induction of insulin resistance may be a physiological adaptation process. Therefore, it is suggested that health care providers should pay more attention to daily nutrition management and physical exercise to reduce the risk of stroke in obese and dyslipidemia patients with T2DM, instead of relying on high doses of insulin and sulfonylurea medications [69]. In addition, higher levels of HbA1c correlated positively with increased stroke risk, especially among patients with HbA1c levels above 9% [70–71]. This underlines the importance of glycemic control in preventing stroke. Although the effect of intensive glycemic control on cardiovascular disease is still controversial, studies have shown that the reduction of HbA1c and the prolongation of intensive glycemic control may have a positive effect on cardiovascular disease [72].

An important finding of this study was that complications arising from T2DM were identified as the most robust indicators of stroke risk. Atherosclerosis, aggravated by T2DM, notably increased the risk of stroke, especially in patients with large artery atherosclerosis [5]. Microvascular complications such as DR, DN, and diabetic neuropathy emerged as significant predictors of future macrovascular diseases. And after adjusting for traditional risk factors, DR and DN are still independent predictors of stroke in patients with T2DM [73–74]. Additionally, AF was identified as a substantial risk factor for stroke in T2DM patients [75]. Macrovascular and microvascular complications of T2DM are the main causes of disability and death in patients. However, Due to the long duration of pre-diabetes in most patients, many patients have macrovascular and microvascular damage before the onset of overt diabetes occurs [61,76]. Therefore, for people with diabetes risk factors and genetic susceptibility, health care personnel should carefully assess their macrovascular and microvascular changes and guide them to follow a healthy lifestyle to prevent or timely detect macrovascular and microvascular complications. In addition, it is deemed essential for future articles to find new predictors such as biomarkers and related gene induction studies [77].

Another finding of this study was MET, Pioglitazone, and MET combination therapy were protective factors against stroke in T2DM patients. The cardiovascular protective effects of MET and pioglitazone have been confirmed in previous studies, but for patients with existing cardiovascular diseases, there is insufficient evidence to rely on monotherapy [76]. One research [78] have shown that MET combination therapy can better control blood glucose, while reducing the risk of late glycemic control failure, and did not increase hypoglycemic events. It appears that our findings differ because the included article in our study compared MET combination therapy with MET + SU, rather than directly contrasting it with MET monotherapy. Otherwise, the American Diabetes Association and the European Association for the Study of Diabetes [79] recommend SGLT2-i or GLP-1RA as hypoglycemic agents for patients at high risk of cardiovascular disease, and studies [11] have shown that SGLT2-i or GLP-1RA may reduce the risk of stroke in patients with T2DM. Nonetheless, given the limited number of articles included, there is not adequate evidence to conclusively support these findings. Consequently, there is a clear indication for additional large-scale prospective studies to validate and further substantiate these conclusions in the future.

In summary, stroke occurrence in T2DM patients is multifactorial, influenced by a spectrum of variables. Beyond conventional pharmacological approaches, the cultivation of enduring healthy habits, including adherence to a well-rounded nutritional regimen, cessation of smoking, and consistent engagement in physical exercise, stands as imperative in averting stroke incidents [79]. As personalized medicine advances, preventing strokes in T2DM necessitates a holistic approach, leveraging accurate personalized risk prediction models powered by algorithms. We anticipate this study to serve as a reference point for enhancing related risk prediction models. Significantly, our analysis solely scrutinized hypoglycemic agents. Hence, to devise a more comprehensive strategy for managing T2DM, there exists an urgent imperative to delve deeper into the interrelationships among diverse medications, including antihypertensive agents, lipid-lowering medications, antiplatelet therapies, and multifaceted drug regimens concerning stroke occurrences in individuals with T2DM. This holistic exploration would markedly enhance our comprehension and fortify therapeutic approaches aimed at addressing the complexities of managing T2DM complications, specifically in the context of reducing the peril associated with strokes.

### 4.2 Strengths and weaknesses

The strengths of our systematic review consist of the included articles are high-quality. Moreover, we specifically analyzed the complications and hypoglycemic medications of T2DM patients. This can offer a more comprehensive reference for the holistic management of stroke risk in T2DM patients. In addition, some potentially modifiable risk factors offers actionable insights into preventive strategies.

However, several limitations in this review merit acknowledgment. Firstly, observational studies inherently carry confounding factors. While we extracted multivariate adjusted OR, the likelihood of other unmeasured factors influencing the actual relationships cannot be dismissed. Secondly, although numerous factors were explored, the limited number of individual studies impedes a comprehensive elucidation of crucial factors contributing to the heterogeneity in research outcomes, such as regional disparities, racial influences, and sample sizes. Furthermore, the predominance of studies from Asian regions raises concerns about the generalizability and representativeness of the results. Moreover, not all articles included were prospective studies, which curtails establishing a definitive causal relationship between outcomes and variables. Hence, the results should be interpreted cautiously, considering these limitations.

## 5. Conclusion

This comprehensive review and meta-analysis identified several prominent risk factors associated with stroke in patients diagnosed with T2DM. Age, gender, T2DM duration, hypertension, dyslipidemia, smoking habits, elevated HbA1c levels, and various T2DM-related complications such as CHD, DR, AF, DN, PVD, and carotid plaque were all identified as significant risk factors. Conversely, exercise, HDL-C, and certain hypoglycemic agents demonstrated a protective effect against stroke in these patients. Healthcare practitioners can leverage these findings to develop targeted prevention strategies for individuals with T2DM. Beyond advocating for lifestyle improvements, proactive screening for both macrovascular and microvascular complications is crucial. Additionally, the judicious adjustment of hypoglycemic medications holds promise in mitigating stroke risk in this patient population.

## Supporting information

S1 Checklist PRISMA checklist. (DOCX)

S1 Appendix (DOCX)
