# Supplementary material for: Influencing factors of stroke in patients with type 2 diabetes: A systematic review and meta-analysis
Source: PLoS One. 2024 Jun 24;19(6):e0305954. doi: 10.1371/journal.pone.0305954 (PMC11196000; doi:10.1371/journal.pone.0305954)
Supplement: S1 Appendix — (DOCX) [file pone.0305954.s002.docx]

# **S1 Appendix**

# S1 Table**:** Search strategy

| **PubMed (Initial search May 2023, updated August 2023)** |
| --- |
| (((("Diabetes Mellitus, Type 2"[Mesh]) OR ((((((((Diabetes Mellitus, Noninsulin-Dependent[Title/Abstract]) OR (Diabetes Mellitus, Non Insulin Dependent[Title/Abstract])) OR (Diabetes Mellitus, Non-Insulin-Dependent[Title/Abstract])) OR (Non-Insulin-Dependent Diabetes Mellitus[Title/Abstract])) OR (Diabetes Mellitus, Type II[Title/Abstract])) OR (Type 2 Diabetes Mellitus[Title/Abstract])) OR (Type 2 Diabetes[Title/Abstract])) OR (Diabetes, Type 2[Title/Abstract]))) AND (("Stroke"[Mesh]) OR ((((((((((((((((Strokes[Title/Abstract]) OR (Cerebrovascular Accident*[Title/Abstract])) OR (Cerebrovascular Apoplexy[Title/Abstract])) OR (Apoplexy, cerebrovascular[Title/Abstract])) OR (Vascular Accident, Brain[Title/Abstract])) OR (Brain Vascular Accident*[Title/Abstract])) OR (Cerebrovascular Stroke*[Title/Abstract])) OR (Apoplexy[Title/Abstract])) OR (Stroke, Acute[Title/Abstract])) OR (Acute Cerebrovascular Accident[Title/Abstract])) OR (Hemorrhagic Stroke*[Title/Abstract])) OR (Ischemic Stroke*[Title/Abstract])) OR (Acute Ischemic Stroke*[Title/Abstract])) OR (Thrombotic Stroke*[Title/Abstract])) OR (Embolic Stroke*[Title/Abstract])) OR (Cerebral Infarction*[Title/Abstract])))) AND (("Risk Factors"[Mesh]) OR (((risk factor[Title/Abstract]) OR (influence factor*[Title/Abstract])) OR (Relevant Factor*[Title/Abstract])))) |
| **Embase (Initial search May 2023, updated August 2023)** |
| ('Diabetes Mellitus, Noninsulin-Dependent':ab,ti OR 'Diabetes Mellitus, Non Insulin Dependent':ab,ti OR 'Diabetes Mellitus, Non-Insulin-Dependent':ab,ti OR 'Non-Insulin-Dependent Diabetes Mellitus':ab,ti OR 'Diabetes Mellitus, Type II':ab,ti OR 'Type 2 Diabetes Mellitus':ab,ti OR 'Type 2 Diabetes':ab,ti OR 'Diabetes, Type 2':ab,ti) AND ('Strokes':ab,ti OR 'Cerebrovascular Accident*':ab,ti OR 'Cerebrovascular Apoplexy':ab,ti OR 'Apoplexy, Cerebrovascular':ab,ti OR 'Vascular Accident, Brain':ab,ti OR 'Brain Vascular Accident*':ab,ti OR 'Cerebrovascular Stroke*':ab,ti OR 'Apoplexy':ab,ti OR 'Stroke, Acute':ab,ti OR 'Acute Cerebrovascular Accident':ab,ti OR 'Hemorrhagic Stroke*':ab,ti OR 'Ischemic Stroke*':ab,ti OR 'Acute Ischemic Stroke*':ab,ti OR 'Thrombotic Stroke*':ab,ti OR 'Embolic Stroke*':ab,ti OR 'Cerebral Infarction*':ab,ti) AND ('risk factor':ab,ti OR 'influence factor*':ab,ti OR 'Relevant Factor*':ab,ti) |
| **Web of Science Core Collection (Initial search May 2023, updated August 2023)** |
| TS=(Diabetes Mellitus, Type 2 OR Diabetes Mellitus, Noninsulin-Dependent OR Diabetes Mellitus, Non Insulin Dependent OR Diabetes Mellitus, Non-Insulin-Dependent OR Non-Insulin-Dependent Diabetes Mellitus OR Diabetes Mellitus, Type II OR Type 2 Diabetes Mellitus OR Type 2 Diabetes OR Diabetes, Type 2) AND TS=(Stroke* OR Cerebrovascular Accident* OR Cerebrovascular Apoplexy OR Apoplexy, Cerebrovascular OR Vascular Accident, Brain OR Brain Vascular Accident* OR Cerebrovascular Stroke* OR Apoplexy OR Stroke, Acute OR Acute Cerebrovascular Accident OR Hemorrhagic Stroke* OR Ischemic Stroke* OR Acute Ischemic Stroke* OR Thrombotic Stroke* OR Embolic Stroke* OR Cerebral Infarction*) AND TS=(risk factor* OR influence factor* OR Relevant Factor*) |
| **The Cochrane Library (Initial search May 2023, updated August 2023)** |
| (Diabetes Mellitus, Noninsulin-Dependent):ti,ab,kw OR (Diabetes Mellitus, Non Insulin Dependent):ti,ab,kw OR (Diabetes Mellitus, Non-Insulin-Dependent):ti,ab,kw OR (Non-Insulin-Dependent Diabetes Mellitus):ti,ab,kw OR (Diabetes Mellitus, Type II):ti,ab,kw OR (Type 2 Diabetes Mellitus):ti,ab,kw OR (Type 2 Diabetes):ti,ab,kw OR (Diabetes, Type 2):ti,ab,kw AND (Strokes):ti,ab,kw OR (Cerebrovascular Accident*):ti,ab,kw OR (Cerebrovascular Apoplexy):ti,ab,kw OR (Apoplexy, Cerebrovascular):ti,ab,kw OR (Vascular Accident, Brain):ti,ab,kw OR (Brain Vascular Accident*):ti,ab,kw OR (Cerebrovascular Stroke*):ti,ab,kw OR (Apoplexy):ti,ab,kw OR (Stroke, Acute):ti,ab,kw OR (Acute Cerebrovascular Accident):ti,ab,kw OR (Hemorrhagic Stroke*):ti,ab,kw OR (Ischemic Stroke*):ti,ab,kw OR (Acute Ischemic Stroke*):ti,ab,kw OR (Thrombotic Stroke*):ti,ab,kw OR (Embolic Stroke*):ti,ab,kw OR (Cerebral Infarction*):ti,ab,kw AND (risk factor):ti,ab,kw OR (influence factor*):ti,ab,kw OR (Relevant Factor*):ti,ab,kw |
| **China National Knowledge Infrastructure (Initial search May 2023, updated August 2023)** |
| (主题=2型糖尿病) AND (主题=脑卒中 + 脑中风 + 脑出血 + 脑缺血 + 脑梗死 + 脑血管意外) AND (主题=危险因素 + 相关因素 + 影响因素) |
| **Chinese Biomedical Literature Database (Initial search May 2023, updated August 2023)** |
| ("糖尿病, 2型"[不加权:扩展] ) AND ("卒中"[不加权:扩展] OR (((("脑出血"[常用字段:智能]) OR "脑缺血"[常用字段:智能]) OR "脑梗死"[常用字段:智能]) OR "脑血管意外"[常用字段:智能]) OR "脑中风"[常用字段:智能]) AND ("危险因素"[不加权:扩展] OR ("相关因素"[常用字段:智能]) OR "影响因素"[常用字段:智能]) |
| **Wanfang database (Initial search May 2023, updated August 2023)** |
| (主题:(2型糖尿病) and 主题:(脑卒中 OR 脑中风 OR 脑出血 OR 脑缺血 OR 脑梗死 OR 脑血管意外) and 主题:(危险因素 OR 相关因素 OR 影响因素) ) |
| **Vip citation database (Initial search May 2023, updated August 2023)** |
| (题名或关键词=2型糖尿病 AND 题名或关键词=中风+脑卒中+缺血性脑卒中+脑缺血+脑出血+脑梗死+脑血管意外 AND 题名或关键词=危险因素+相关因素+影响因素) |

S2 Table: Results of quality assessment of cohort studies on NOS

| **Study** | **Selection** | | | | **Comparability** | | **Exposure/Outcome** | | **score** | |  |
| --- | --- | --- | --- | --- | --- | --- | --- | --- | --- | --- | --- |
|  | **1** | **2** | **3** | **4** | **5A** | **5B** | **6** | **7** | **8** |  | |
| Yu,2023 | 1 | 1 | 1 | 1 | 1 | 1 | 1 | 0 | 1 | 8 | |
| Zhou,2022 | 1 | 1 | 1 | 1 | 1 | 1 | 1 | 1 | 1 | 9 | |
| Wu,2022 | 1 | 1 | 1 | 1 | 1 | 1 | 1 | 1 | 1 | 9 | |
| Kim,2022 | 1 | 1 | 1 | 1 | 1 | 1 | 1 | 1 | 1 | 9 | |
| Lin,2022 | 1 | 1 | 1 | 1 | 1 | 1 | 0 | 1 | 1 | 8 | |
| Iwase,2021 | 1 | 1 | 1 | 1 | 0 | 1 | 1 | 1 | 1 | 8 | |
| Isaman,2021 | 1 | 0 | 1 | 1 | 1 | 1 | 1 | 1 | 1 | 8 | |
| Salinero-Fort,2021 | 1 | 1 | 1 | 1 | 1 | 1 | 1 | 1 | 1 | 9 | |
| Modjtahedi,2021 | 1 | 1 | 1 | 1 | 1 | 1 | 1 | 1 | 1 | 9 | |
| Drinkwater,2020 | 1 | 1 | 1 | 1 | 1 | 1 | 1 | 1 | 1 | 9 | |
| Kim,2020 | 1 | 1 | 1 | 1 | 1 | 1 | 1 | 1 | 1 | 9 | |
| Adderley,2020 | 1 | 1 | 1 | 1 | 1 | 1 | 1 | 1 | 1 | 9 | |
| Fangel,2020 | 1 | 1 | 1 | 0 | 1 | 1 | 1 | 1 | 1 | 8 | |
| Hung,2020 | 1 | 1 | 1 | 0 | 0 | 1 | 1 | 1 | 1 | 7 | |
| Komi,2018 | 1 | 1 | 1 | 1 | 1 | 1 | 1 | 1 | 1 | 9 | |
| Chan,2018 | 1 | 1 | 1 | 1 | 1 | 1 | 1 | 1 | 1 | 9 | |
| Hsu,2018 | 1 | 1 | 1 | 0 | 1 | 1 | 1 | 0 | 1 | 7 | |
| Noh,2017 | 1 | 1 | 1 | 1 | 1 | 1 | 1 | 0 | 1 | 8 | |
| Ou,2017 | 1 | 1 | 1 | 0 | 1 | 1 | 1 | 1 | 1 | 8 | |
| Zimmerman,2017 | 1 | 1 | 1 | 1 | 1 | 1 | 1 | 1 | 1 | 9 | |
| Zghebi,2016 | 1 | 1 | 1 | 0 | 1 | 1 | 1 | 0 | 1 | 7 | |
| Liu,2014 | 1 | 1 | 1 | 1 | 1 | 1 | 1 | 0 | 1 | 8 | |
| Wang,2014 | 1 | 0 | 1 | 1 | 1 | 1 | 1 | 1 | 1 | 8 | |
| Li,2014 | 1 | 1 | 1 | 1 | 1 | 1 | 1 | 0 | 1 | 8 | |
| Cheng,2014 | 1 | 1 | 1 | 1 | 1 | 1 | 1 | 0 | 1 | 8 | |
| Marfella,2013 | 1 | 1 | 1 | 1 | 1 | 1 | 1 | 0 | 1 | 8 | |
| Elley,2008 | 1 | 1 | 1 | 1 | 1 | 1 | 1 | 1 | 1 | 9 | |
| Gillett,2003 | 1 | 1 | 1 | 1 | 1 | 1 | 1 | 1 | 1 | 9 | |

S3 Table: Results of quality assessment of case-control studies on NOS

| **Study** | **Selection** | | | | **Comparability** | | **Exposure/Outcome** | | | **score** |
| --- | --- | --- | --- | --- | --- | --- | --- | --- | --- | --- |
|  | **1** | **2** | **3** | **4** | **5A** | **5B** | **6** | **7** | **8** |  |
| Wu2,2022 | 1 | 1 | 1 | 1 | 1 | 1 | 1 | 1 | 1 | 9 |
| Ha,2021 | 1 | 1 | 1 | 1 | 1 | 1 | 1 | 1 | 1 | 9 |
| Su,2020 | 1 | 1 | 1 | 0 | 1 | 1 | 1 | 1 | 1 | 8 |
| Geng,2019 | 1 | 1 | 1 | 1 | 1 | 1 | 1 | 1 | 1 | 9 |
| Niwa,2019 | 1 | 1 | 1 | 1 | 1 | 1 | 0 | 1 | 1 | 8 |
| Ye,2016 | 1 | 1 | 1 | 1 | 1 | 1 | 1 | 1 | 1 | 9 |
| Xu,2004 | 1 | 1 | 1 | 1 | 1 | 1 | 1 | 1 | 1 | 9 |
| Meng,2001 | 1 | 0 | 1 | 1 | 1 | 1 | 1 | 1 | 0 | 7 |

# S4 Table: Results of quality assessment of cross-sectional studies on the criteria of AHRQ

| **Study** | **1** | **2** | **3** | **4** | **5** | **6** | **7** | **8** | **9** | **10** | **11** | **score** |
| --- | --- | --- | --- | --- | --- | --- | --- | --- | --- | --- | --- | --- |
| Xu,2022 | 1 | 1 | 1 | 1 | 1 | 1 | 0 | 1 | 0 | 1 | 0 | 8 |
| He,2021 | 1 | 1 | 1 | 1 | 1 | 1 | 1 | 1 | 0 | 1 | 0 | 9 |
| Shi,2020 | 1 | 1 | 1 | 1 | 1 | 1 | 1 | 1 | 1 | 1 | 0 | 10 |
| Alramadan,2019 | 1 | 0 | 1 | 1 | 1 | 1 | 1 | 1 | 0 | 1 | 0 | 8 |
| Sun,2017 | 1 | 1 | 1 | 1 | 1 | 0 | 1 | 1 | 0 | 1 | 0 | 8 |
| Bouchi,2012 | 1 | 1 | 1 | 1 | 1 | 1 | 0 | 1 | 0 | 1 | 0 | 8 |
| Nomura,2010 | 1 | 0 | 1 | 1 | 1 | 1 | 1 | 1 | 0 | 1 | 0 | 8 |

# S1 Fig: The sensitivity analysis plots with meta-analysis

# **
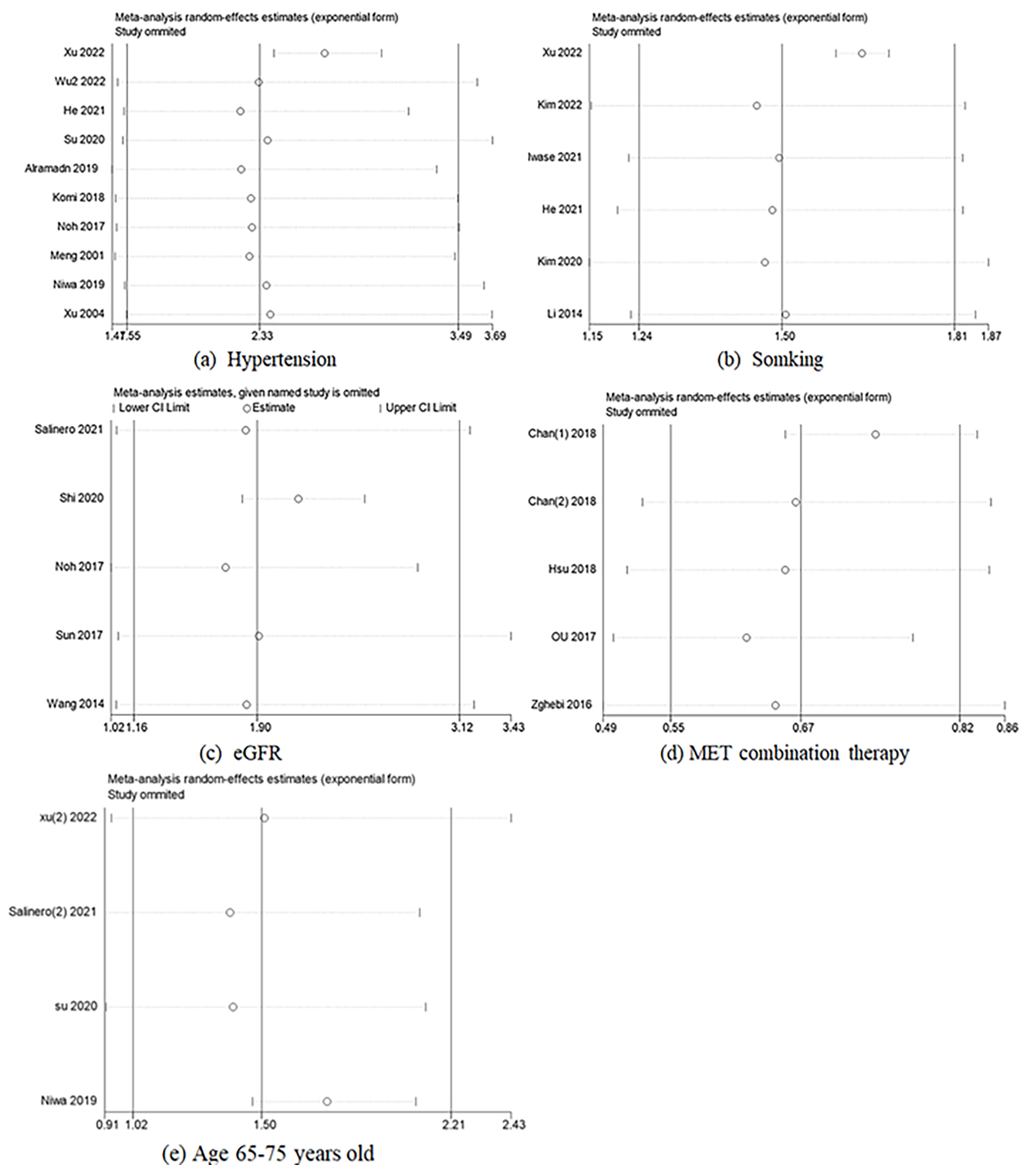
**

# S2 Fig: The forest plot of sociodemographic factors

# **
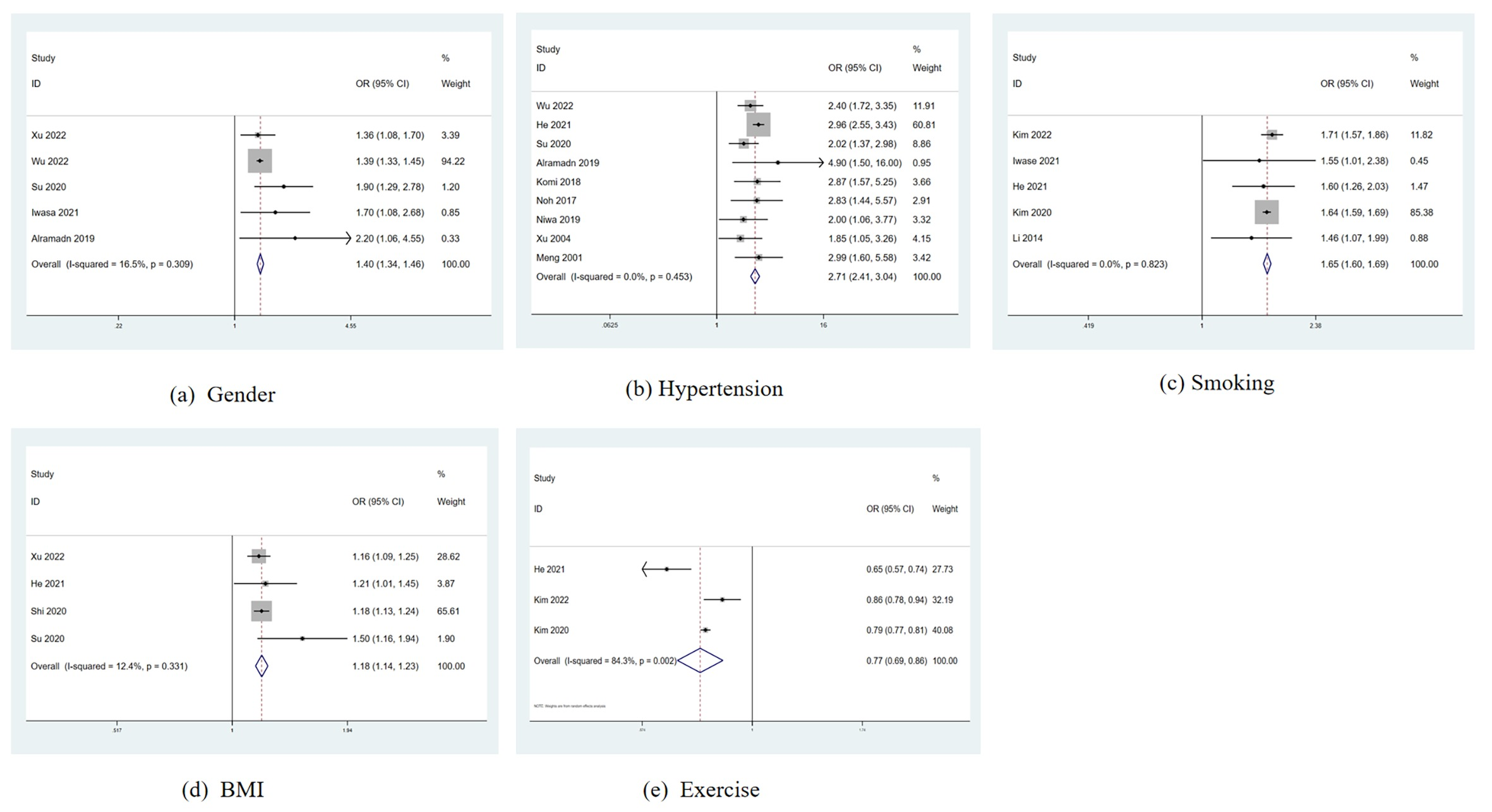
**

[S3 Fig: The forest plot of biochemical factors](#_Toc134190903)

**
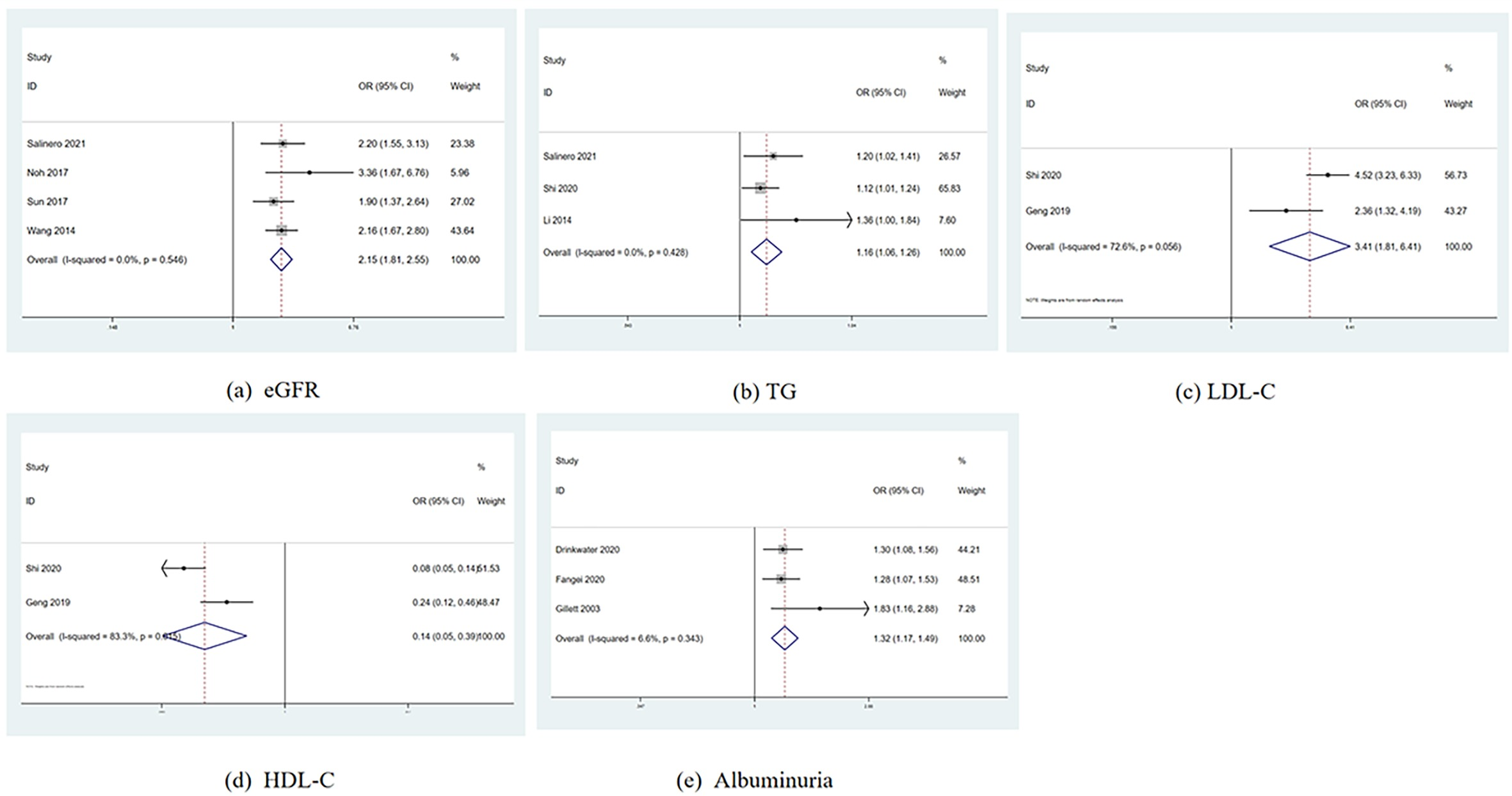
**

[S4 Fig: The forest plot of complications](#_Toc134190904)

**
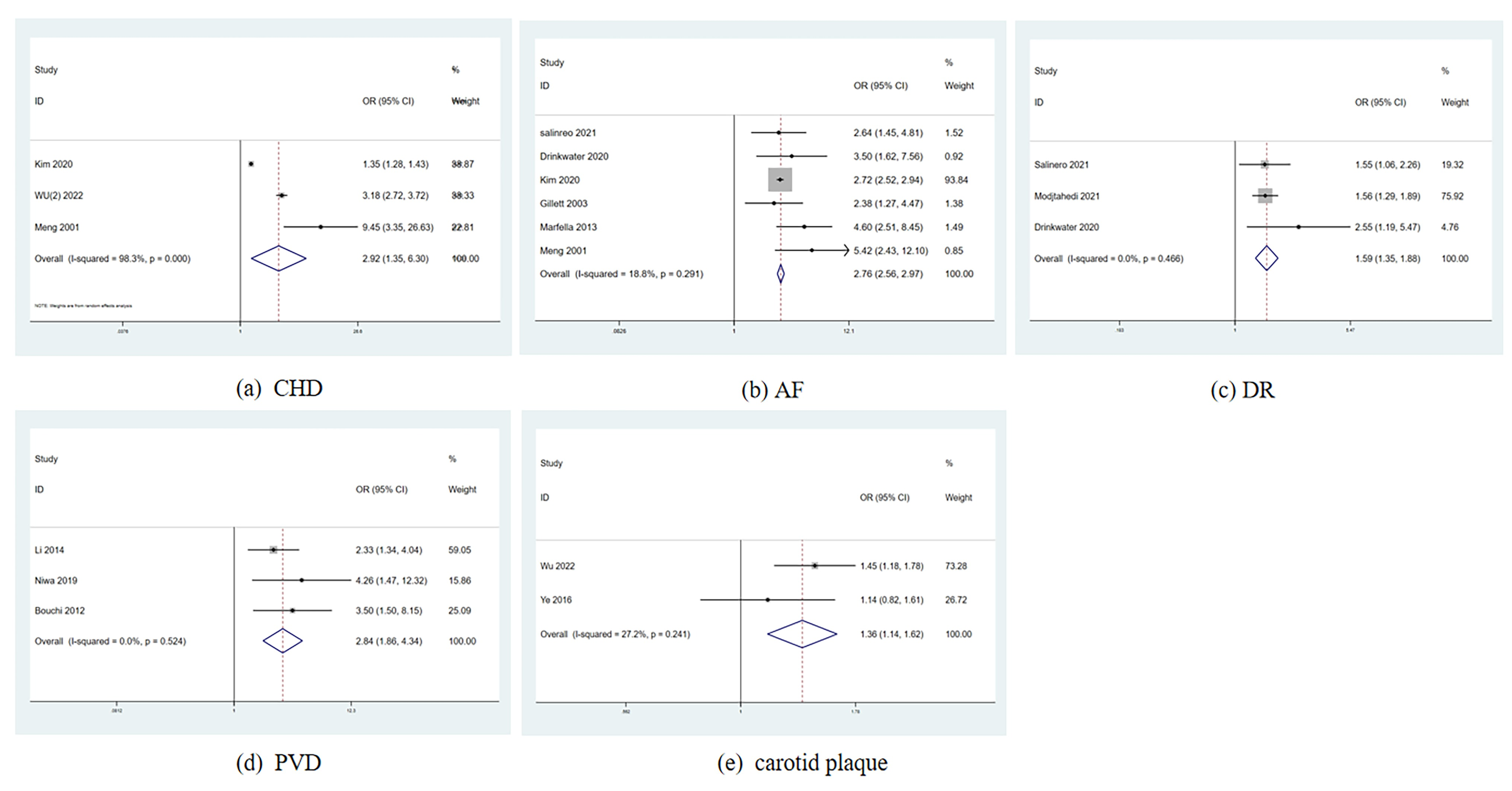
**

[S5 Fig: The forest plot of hypoglycemic agents](#_Toc134190905)

**
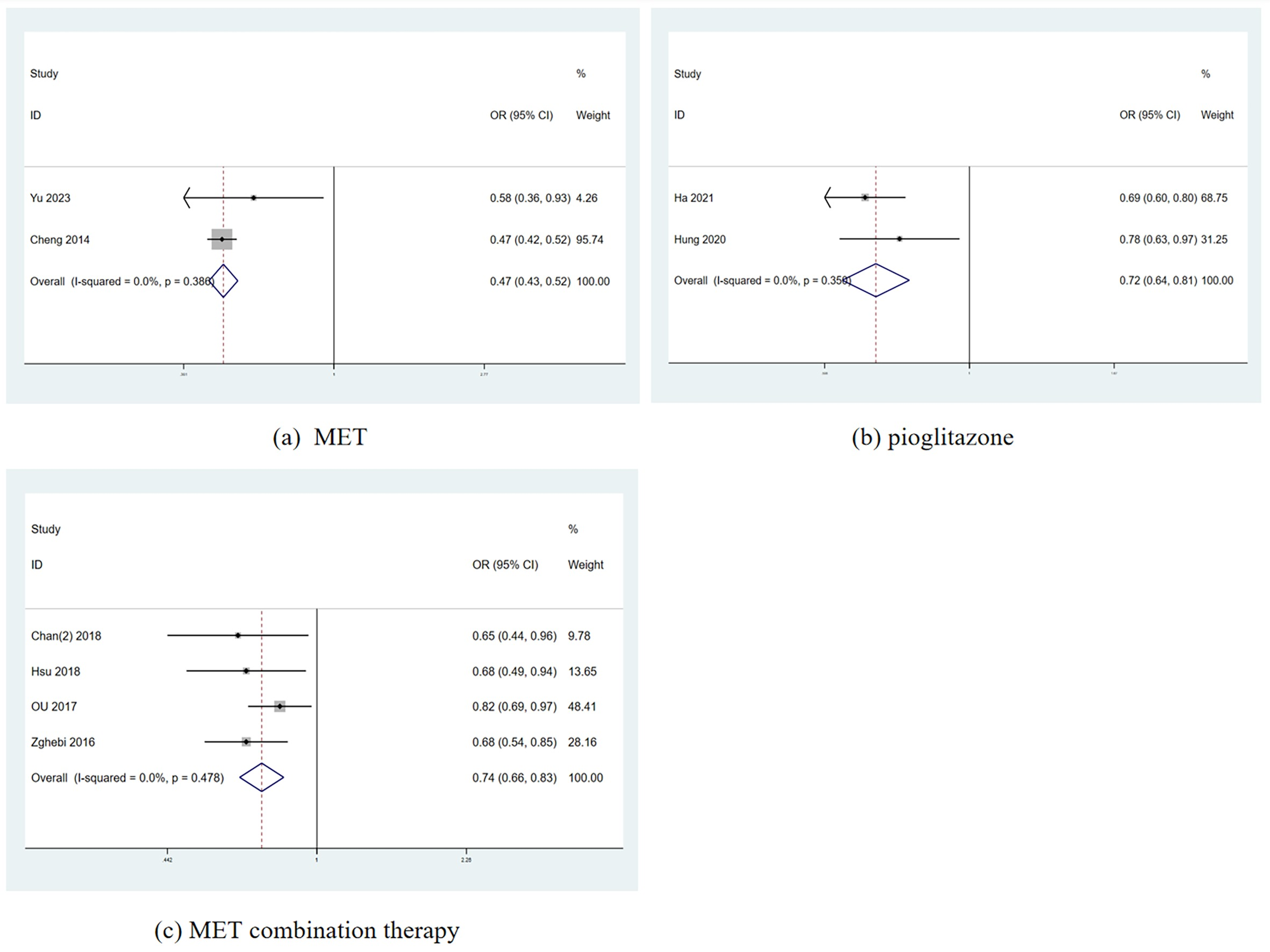
**
